# Thiopurine *S*‑methyltransferase- and indolethylamine *N*‑methyltransferase-mediated formation of methylated tellurium compounds from tellurite

**DOI:** 10.1007/s00204-024-03890-4

**Published:** 2024-10-17

**Authors:** Yu-ki Tanaka, Ayuka Takata, Karin Takahashi, Yoshikazu Yamagishi, Yasunori Fukumoto, Noriyuki Suzuki, Yasumitsu Ogra

**Affiliations:** 1 Graduate School of Pharmaceutical Sciences, Chiba University, 1-8-1 Inohana, Chuo, Chiba 260-8675 Japan; 2 Graduate School of Medicine, Chiba University, 1-8-1 Inohana, Chuo, Chiba 260-8670 Japan; 3 Present Address: Faculty of Pharmaceutical Sciences, Toho University, 2-2-1 Miyama, Funabashi, Chiba 274-8510 Japan

**Keywords:** INMT, LC-ICP-MS, Methylation, Tellurium, TPMT

## Abstract

Tellurium (Te) is a metalloid widely used in various industries. However, its toxicological impact on humans is poorly understood. In this study, we investigated the role of two methyltransferases, thiopurine *S*‑methyltransferase (TPMT) and indolethylamine *N*‑methyltransferase (INMT), in the methylation of tellurite, an inorganic Te oxyanion. The products of the reaction of Te compounds catalyzed by recombinant human TPMT and/or INMT were analyzed by liquid chromatography hyphenated to inductively coupled plasma mass spectrometry (LC-ICP-MS) and gas chromatography mass spectrometry (GC-MS). We found that TPMT catalyzes the methylation of non-methylated Te and methanetellurol to generate dimethyltelluride. On the other hand, INMT catalyzes the methylation of methanetellurol and dimethyltelluride to produce trimethyltelluronium ion, a metabolite excreted into animal urine. We conclude that TPMT and INMT are cooperatively responsible for the detoxification of Te oxyanions through methylation to form trimethyltelluronium ions.

**Supplementary Information:**

The online version contains supplementary material available at 10.1007/s00204-024-03890-4.

## Introduction

Tellurium (Te) is widely used in various industries owing to its unique physicochemical properties as a metalloid (Medina-Cruz et al. 2020). Similar to oxygen, sulfur, and selenium (Se), Te is a chalcogen element. However, unlike the other chalcogens, Te is a non-essential element in animals. The toxicological impact of Te on human health and the environment has been extensively investigated. There are reports that inorganic Te salt tellurate is metabolized to form organotellurium compounds in higher plants (Anan et al. 2013; Cowgill 1988; Matos Reyes et al. 2009). For instance, garlic (*Allium sativum*), a chalcogen hyperaccumulator (Ogra et al. 2015), produces three Te-containing metabolites (Anan et al. 2013; Ogra et al. 2015), two of which were identified as *Te*-methyltellurocysteine (a telluroamino acid) and *S*-methyltellurosulfide. The third Te-containing metabolite in garlic, which is also found in Indian mustard (*Brassica juncea*) (Ogra et al. 2010), has recently been identified as gluconic acid-3-tellurate (Takada et al. 2024). In addition to the synthesis of telluroamino acids, the production of insoluble elemental Te particles may be a critical pathway for Te detoxification in plants and microorganisms (Baesman et al. 2007; Borsetti et al. 2003; Di Tomaso et al. 2002; Takada et al. 2022; Tanaka et al. 2020; Yurkov et al. 1996). We have recently reported the formation of insoluble elemental Te nanorods (i.e., biogenic Te nanorods) in mammalian cell culture (Tanaka et al. 2024). However, little is known about the metabolism of Te in animals.

In animals, the alkylation of metals and metalloids is intertwined with the modification of their toxicity. Alkylated forms of mercury and tin are highly toxic to animals compared with their non-alkylated forms (Filonov et al. 2018; Lohren et al. 2015). The stepwise methylation of inorganic arsenic (As), arsenite, catalyzed by arsenite methyltransferase (AS3MT) furnishes dimethylarsinous acid and ultimately trimethylarsine (Lin et al. 2002). Since monomethylated and dimethylated As are excreted via urine, methylation can contribute to reducing As toxicity in the animal body (Crecelius 1977; Tam et al. 1979). We have shown that inorganic Se (i.e., selenite) is methylated through cooperative enzymatic reactions catalyzed by thiopurine *S*‑methyltransferase (TPMT) and indolethylamine *N*‑methyltransferase (INMT) (Fukumoto et al. 2020) to yield the final product trimethylselenonium ion, which is usually found in the urine of selenite-exposed animals (Byard 1969). In the same manner as selenite, trimethyltelluronium ion is detected as a predominant Te metabolite in urine samples from tellurite-fed rats (Ogra et al. 2007). In addition, Te is also found in red blood cells as dimethylated Te (Ogra et al. 2008). These results suggest that dimethylated Te in the blood is transported to the kidney, where it is further methylated to form trimethyltelluronium ion, and then excreted in the urine. Thus, the methylation of Te may be an important biological pathway for reducing Te toxicity. We revealed that the knockdown of the TPMT gene decreases the resistance to tellurite as well as selenite in mammalian cell culture (Fukumoto et al. 2020). From these results, we considered that both TPMT and INMT have the potential to metabolize inorganic Te into methylated Te compounds, which are less toxic excretory products in animals.

In this study, we investigated the role of TPMT and INMT in the methylation of Te compounds using human recombinant proteins produced in *Escherichia coli* (*E. coli*). After the enzymatic reaction, Te compounds were separately detected by liquid chromatography hyphenated to inductively coupled plasma mass spectrometry (LC-ICP-MS). On the basis of the results obtained, we discuss TPMT- and INMT-mediated detoxification of Te oxyanions through the methylation of inorganic Te.

## Experimental

### Reagents

Potassium tellurite was purchased from FUJIFILM Wako Pure Chemical Corporation (Osaka, Japan). Dimethyl ditelluride, the source of methanetellurol, was purchased from Tokyo Chemical Industry Co., Ltd. (Tokyo, Japan). Since there was no commercially available reagent for dimethyltelluride and trimethyltelluronium ion, we synthesized these methylated Te compounds as described in the following section. *S*-Adenosyl methionine (SAM) as a methyl group donor was purchased from New England Biolabs Japan, Inc. (Tokyo, Japan). Reduced glutathione (Nacalai Tesque, Inc., Kyoto, Japan) was used as a reducing agent to generate telluride and/or tellurodiglutathione, a potential substrate for TPMT, following a previous study of selenium (Fukumoto et al. 2020). Hydrogen peroxide (FUJIFILM Wako Pure Chemical Corporation) was used to transform volatile Te compounds, such as methanetellurol and dimethyltelluride, into less volatile oxidized forms. Milli-Q water with a specific resistance of 18.2 MΩ·cm was used throughout the experiments (Merck Millipore, Burlington, MA, USA).

### Synthesis of methylated Te compounds

Dimethyltelluride was prepared from elemental Te following the method described below. Powdered elemental Te (Strem, Newburyport, MA, USA) was suspended in four equivalents of methyl iodide (Nacalai Tesque, Inc.) and the mixture was heated at 80 °C for 48 h. Elemental Te was completely dissolved after heating, and a red precipitate was produced. The precipitate was collected by washing with chloroform (Nacalai Tesque, Inc.) to remove residual methyl iodide. For trimethylated form, we used trimethyltelluronium iodide synthesized and characterized in our previous study (Ogra et al. 2008).

### Expression and purification of recombinant human TPMT and INMT

Recombinant human TPMT and INMT were expressed in *Escherichia coli* (*E. coli*). pBAD vectors with the cDNA sequence of hTPMT were transfected into TOP10 *E. coli* and pET29b vectors with the cDNA sequence of hINMT were transfected into BL21(DE3) *E. coli* by electroporation. The cells were cultured in an LB medium until the optical density at 600 nm (OD600) reached 0.5. Protein expression was induced by the addition of L-arabinose (FUJIFILM Wako Pure Chemical Corporation) for TPMT and isopropyl β-D-1-thiogalactopyranoside (IPTG, Takara Bio Inc., Shiga, Japan) for INMT. After induction for 4 h at 37 °C, the cells were collected by centrifugation. The cells were suspended in a lysis buffer (20 mM sodium phosphate, 300 mM NaCl), subjected to three freeze–thaw cycles using liquid nitrogen, and ultrasonicated 10 times at 10 s each (5 W, 28 kHz, TOMY SEIKO CO., LTD., Tokyo, Japan). To extract the proteins, 0.1% Triton X-100 was added, and the lysate was cleared by centrifugation. The histidine-tagged TPMT and INMT proteins were purified with TALON resin (Takara Bio Inc.) and finally recovered with elution buffer (20 mM sodium phosphate, 300 mM NaCl, 300 mM imidazole, 0.1% Triton X-100, pH 6.8). The collected fractions were dialyzed with a Slide-A-Lyzer Dialysis Cassette (molecular weight cutoff 10 kDa; Thermo Fisher Scientific, Waltham, MA, USA) using a dialysis buffer (20 mM Tris–HCl, 100 mM NaCl, 0.1% Triton X-100, pH 7.4) for 24 h.

### Reaction of Te compounds catalyzed by TPMT and INMT

Purified TPMT and INMT were added into a test tube containing Te compounds. The reaction mixture had the following composition: 40 μg/mL TPMT and/or INMT, 1.0 μM SAM, 10 mM reduced glutathione, and 20 mM sodium phosphate buffer (pH 6.8). Te compounds potassium tellurite, dimethyl ditelluride, and dimethyltelluride were used as substrates at the concentration of 2.0 μM. The reaction was allowed to proceed at 37 °C for 24 h. This was followed by incubation with 3% hydrogen peroxide for another 30 min. As noted above, volatile Te species, such as methanetellurol and dimethyltelluride, were converted into their oxidized forms by hydrogen peroxide. Excess hydrogen peroxide was degraded with 0.1 μg/mL catalase at 37 °C for 1 h. The mixture was filtered through a 0.45 μm PTFE syringe-driven filter (Millex-LH, Merck Millipore), and the filtrate was analyzed by LC-ICP-MS.

### Evaluation of methylated compounds by LC-ICP-MS

An HPLC system was used (Prominence; Shimadzu, Kyoto, Japan) in combination with an anion-exchange column (PRP-X 100, 2.1 × 250 mm, 5 μm particle size; Hamilton, Reno, NV, USA) for the separation of Te compounds. A 20 μL aliquot of the reaction mixture was applied to the column, and the column was eluted with 50 mM Tris-HCl (pH 8.2) at the flow rate of 0.4 mL/min. The eluate from HPLC was continuously introduced into an ICP-MS (Agilent 8800 ICP-MS/MS; Agilent Technologies, Tokyo, Japan). Te signal intensities was monitored at *m/z* 126, 128, and 130 with an integration time of 0.1 s each. The signal intensity of ^128^Te was used in this study. Details of instrument settings and operation conditions are summarized in Table 1.

**Table 1:** Instrumentation and operational settings

HPLC system	
Instrument	Prominence (Shimadzu)
Column	PRP-X 100
Eluent flow rate	0.4 mL/min
Eluent	Tris-HCl (pH 8.2)
Sample volume	20 μL
ICP-MS	
Instrument	Agilent 8800 (Agilent Technologies)
ICP incident power	1600 W
Cooling gas flow rate	15.0 L/min
Auxiliary gas flow rate	0.80 L/min
Nebulizer gas flow rate	1.0 L/min
Collision cell	Disabled
Integration time	0.1 s
Monitored isotope	^126^Te, ^128^Te, ^130^Te
GC-MS	
Instrument	5975C GC/MSD (Agilent Technologies)
Column	DB-5 ms
Injector temperature	280℃
Injection flow	50 mL/min for 2 min (splitless)
Carrier gas	1.0 L/min
Oven temperature	35–200℃ (5℃/min)
Monitored *m/z*	154, 155, 156, 158, 160
Dwell time	70 μs

### Detection of volatile methylated compounds by GC-MS

We confirmed the enzymatic formation of dimethylated Te compounds by gas chromatography–mass spectrometry (GC-MS) because methanetellurol and dimethyltelluride could not be separately detected by LC-ICP-MS. Following the method noted above, the reaction of potassium tellurite was performed with or without TMPT in a glass crimp vial at 37 °C for 24 h. Solid-phase microextraction (SPME) fiber (Carboxen/Polydimethylsiloxane, d_f_ 75 μm, 24 gauge; SUPELCO, Bellefonte, PA, USA) was inserted into the rubber cap to collect volatile dimethyltelluride in the headspace of the vial during the incubation. The vial was heated at 50 °C for 2 h to completely volatilize dimethyltelluride in the reaction mixture. The collected volatile sample that adsorbed to the SPME fiber was injected into GC-MS with a capillary column, DB-5 ms (30 m × 0.25 mm i.d., d_f_ 0.25 µm; Agilent J&W, Santa Clara, CA, USA). GC-MS analysis was performed using a mass selective detector coupled to a gas chromatograph (5975C GC/MSD; Agilent Technologies). Signal intensities were monitored at *m/z* 154, 155, 156, 158, and 160, which corresponded to the *m/z* of dimethyltelluride with different Te isotopes (i.e., ^124^Te, ^125^Te, ^126^Te, ^128^Te, and ^130^Te). Details of instrument settings and operation conditions for GC-MS are summarized in Table 1.

### Assay for methyltransferase activity

To evaluate the contribution of TPMT to the methylation of tellurite and methanetellurol, methyltransferase activity was quantitatively evaluated with an MTase-Glo Methyltransferase Assay Kit (Promega, Madison, WI, USA). Purified TPMT was reacted with potassium tellurite or dimethyl ditelluride (methanetellurol) in plastic tubes in the presence of 50 μM SAM, 5 mM reduced glutathione, and 20 mM sodium phosphate buffer (pH 6.8). The concentrations of Te compounds were 0, 0.83, 8.3, and 83 μM for tellurite and 0, 1.52, 15.2, and 152 μM for methanetellurol. After the reaction for 30 min at 37 °C, 1 µL of 6 × MTase-Glo Reagent was added to 5 µL of the reaction mixture, and the mixture was incubated for 30 min at 37 °C to convert *S*-adenosylhomocysteine into ADP. Finally, 6 µL of MTase-Glo Detection Solution was added, and the mixture was incubated for another 30 min at 37 °C to convert ADP into ATP. The amount of ATP was determined from the luminescence intensity of luciferase using a plate reader, SpectraMax iD3 (Molecular Devices, San Jose, CA, USA).

## Results and discussion

### Speciation analysis of Te compounds by LC-ICP-MS

**Fig. 1:**
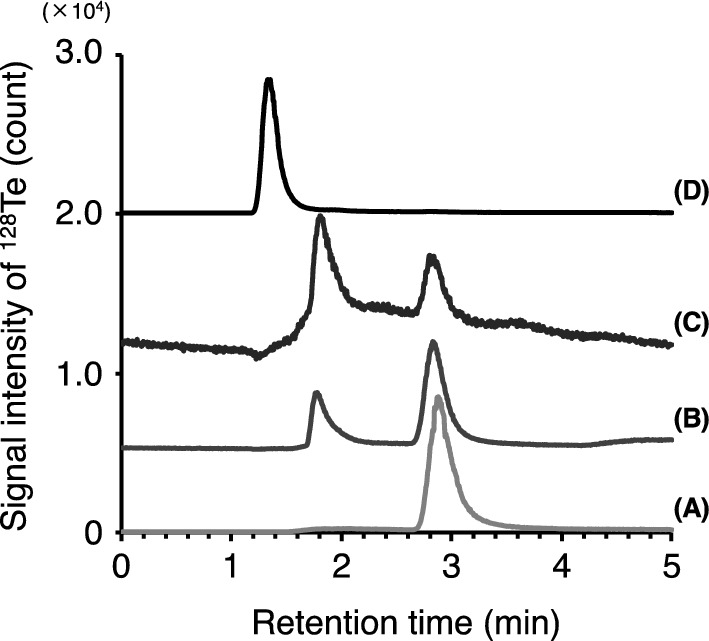
Elution profiles of Te compounds by LC-ICP-MS. Elution profiles of four Te compounds: **A** tellurite, **B** dimethyl ditelluride, **C** dimethyltelluride, and **D** trimethyltelluronium ion treated initially with an enzyme-free reaction mixture and then with hydrogen peroxide and catalase. Since these compounds were oxidized by hydrogen peroxide, the detected peaks in **A**–**C** were derived from tellurate (2.9 min), methanetelluronic acid (1.7 min), and dimethyl telluroxide (1.7 min), respectively. The baseline was manually offset to clarify the signal profiles of each sample

In this study, we developed a method for Te speciation analysis by LC-ICP-MS, which can separately detect Te compounds including tellurite (M_0_), methanetellurol (M_1_), dimethyltelluride (M_2_), and trimethyltelluronium ion (M_3_). We used Te standards treated initially with an enzyme-free reaction mixture and then with hydrogen peroxide and catalase. As shown in Fig. 1, the peaks corresponding to tellurite, methanetellurol, and trimethyltelluronium ion appeared at the different retention times of 2.9 min, 1.7 min, and 1.4 min, respectively. The peak of tellurite detected at the retention time of 2.9 min is consistent with that of tellurate ion (Fig. S1). Hence, tellurite is oxidized by hydrogen peroxide. Indeed, there was almost no peak in the elution profile of tellurite solution without hydrogen peroxide treatment (Fig. S1). In the same manner as tellurite, both methanetellurol and dimethyltelluride were detected by LC-ICP-MS analysis only after oxidation to methanetelluronic acid and dimethyl telluroxide, respectively. The peak corresponding to tellurite (i.e., tellurate) was also detected from the methanetellurol solution. This can be explained by the chemical degradation of methanetellurol by hydrogen peroxide. Chemically synthesized dimethyltelluride sample was eluted at almost the same retention time as that for methanetellurol, and a small amount of tellurite was detected as well as methanetellurol. Thus, we were unable to separately detect methanetellurol (i.e., methanetelluronic acid) and dimethyltelluride (i.e., dimethyl telluroxide) by LC-ICP-MS. We examined the formation of methanetellurol and dimethyltelluride in the enzymatic reaction by GC-MS.

### Enzymatic reaction of tellurite with TPMT and INMT

**Fig. 2:**
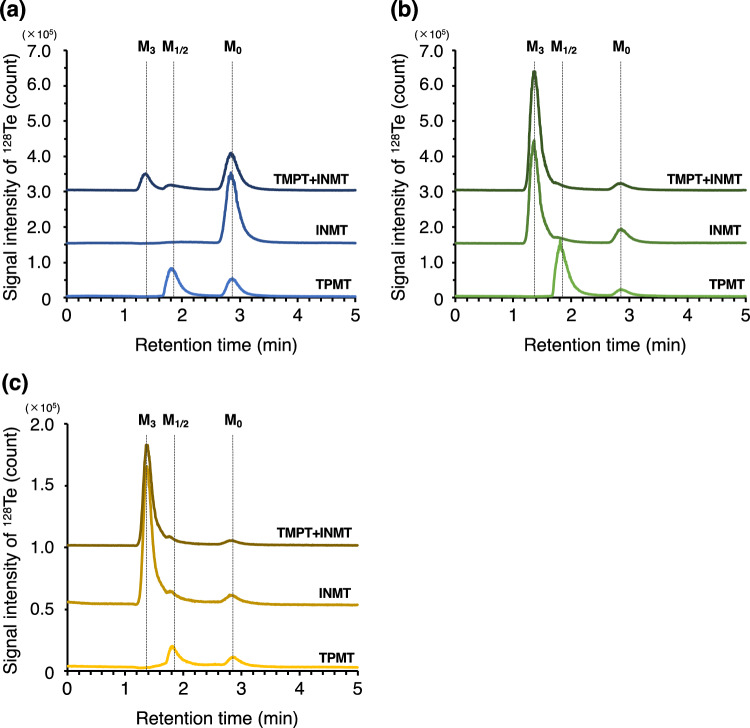
Elution profiles of the reaction mixture with TPMT and/or INMT by LC-ICP-MS. **a** Tellurite, **b** dimethyl ditelluride (monomethylated form), and **c** dimethyltelluride (dimethylated form) were used as substrates. The baseline was manually offset to clarify the signal profiles of each sample

**Fig. 3:**
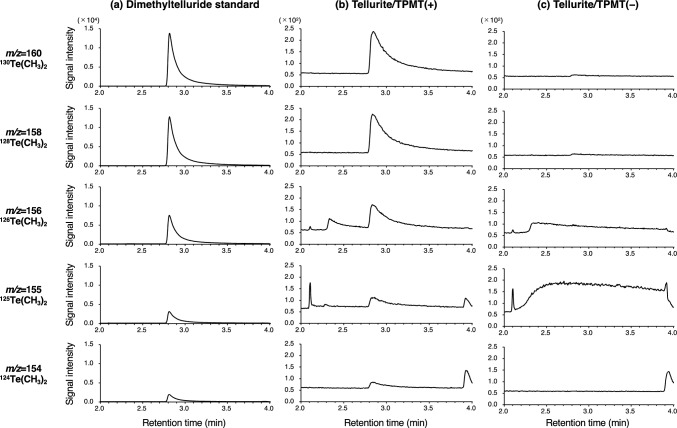
Chromatograms derived from GC-MS analysis at *m/z* 154, 155, 156, 158, and 160. **a** Dimethyltelluride standard, **b** reaction mixture of tellurite with TPMT, and **c** reaction mixture of tellurite without TPMT

Enzymatic reactions were carried out using tellurite, dimethyl ditelluride, and dimethyltelluride as substrates. The elution profiles are shown in Fig. 2. When tellurite was incubated with TPMT, the peak of M_1_ and/or M_2_ was detected, but that of M_3_ was not. To confirm the formation of M_2_ in the reaction mixture containing TPMT, GC-MS analysis was carried out. As shown in Fig. 3, the signals of dimethyltelluride were detected at *m/z* 154, 155, 156, 158, and 160, which contains ^124^Te, ^125^Te, ^126^Te, ^128^Te, and ^130^Te, respectively. The signal intensities reflected the natural isotopic abundance of Te (Table 2). From this, we concluded that dimethyltelluride was enzymatically generated from tellurite by TPMT. In contrast, no M_1_, M_2_, or M_3_ peak was detected in the elution profile of the reaction mixture of tellurite and INMT. On the other hand, the M_3_ peak was detected in the elution profile of the reaction mixture containing tellurite and both TPMT and INMT.

**Table 2:** Comparison of natural isotopic abundance of Te and the relative peak area of dimethyltelluride detected by GC-MS

*m/z*	Te isotope	Abundance (%)	Relative abundance	Dimethyltelluride		Tellurite/TPMT( +)	
				Peak area	Relative peak area	Peak area	Relative peak area
154	^124^Te	4.74	0.14	260.31	0.14	6.04	0.12
155	^125^Te	7.07	0.21	402.75	0.22	10.62	0.22
156	^126^Te	18.84	0.55	989.36	0.53	25.09	0.52
158	^128^Te	31.74	0.93	1733.51	0.94	45.22	0.93
160	^130^Te	34.08	1.00	1853.40	1.00	48.60	1.00

We were unable to detect clear changes in the elution profile for the enzymatic reaction of dimethyl ditelluride with TPMT. However, we noted that the peak at the retention time of 1.8 min was sharper than the peak derived from the standard solution of dimethyl ditelluride (M_1_), suggesting that M_1_ was partly transformed into M_2_ (Fig. S2). This was supported by the results of GC-MS analysis that showed TPMT contributing to the methylation of tellurite to dimethyltelluride through the formation of dimethyl ditelluride (Fig. 3). The M_3_ peak was detected in the elution profile of the reaction mixture containing INMT. This implies that INMT is involved in the two-step methylation of methanetellurol to form trimethyltelluronium ion. Finally, when dimethyltelluride was used as the substrate, the M_3_ peak was detected in the elution profile of the reaction mixture containing INMT, in the same manner as the methylation of dimethyl ditelluride.

We evaluated the methyltransferase activity of TPMT using tellurite and dimethyl ditelluride as substrates. The resulting Michaelis–Menten plots are shown in Fig. S3, and *K*_m_ and *V*_max_ values are listed in Table 3. By comparing the *K*_m_ and *V*_max_ values of these two substrates, we clarified that TPMT contributed to the methylation of tellurite into methanetellurol more strongly than to the methylation of methanetellurol into dimethyltelluride. When the methylation reaction was performed in the absence of glutathione, no M_1_/M_2_ peak was detected from the reaction mixture of tellurite with TPMT (Fig. S4). In addition, we recently reported that the active site of TPMT preferentially recognizes negatively charged substrates (Fukumoto et al. 2024). Based on these finding, negatively charged glutathionyl telluride anion (GSTe^−^) instead of tellurite (HTeO_3_^−^) can be a potential substrate for TPMT at neutral pH in vivo. The higher p*K*a of methanetellurol than that of glutathionyl telluride might result in a decrease in the amount of the negatively charged (i.e., deprotonated) form of methanetellurol. As a result, the *K*_m_ of TPMT for methanetellurol would be higher than that for tellurite. However, it should be noted that the two-step methylation of tellurite to produce dimethyl ditelluride occurred in the reaction mixture of tellurite and TPMT. On the other hands, INMT preferentially recognizes substrates with neutral charge (Fukumoto et al. 2024). Therefore, protonated methanetellurol (CH_3_TeH) and glutathionyl methanetellurol (GSTeCH_3_) can be a substrate for INMT as well as dimethyltelluride.

**Table 3:** Quantitative analyses of methyltransferase activity of TPMT

Substrate	*V*_max_ (nmol/min∙mg)	*K*_m_ (μM)
Tellurite	1.65 ± 0.24	3.5 ± 1.2
Methanetellurol	1.24 ± 0.19	10.9 ± 1.8

**Scheme 1:**
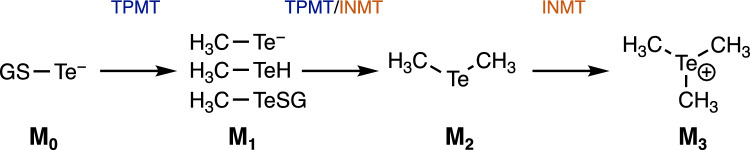
Involvement of TPMT and INMT in the methylation of Te compounds

The results obtained in this study support our scenario that TPMT and INMT can cooperatively catalyze the methylation of Te compounds, similar to the methylation of selenium compounds (Scheme 1). We have already reported that TPMT and INMT cooperatively metabolized selenium to form the ultimate methylated form, trimethylselenonium ion (Fukumoto et al. 2020, 2024). Taken together with the results obtained in this study, TPMT and INMT was unable to distinguish tellurium and selenium to form their trimethylated forms. In this study, we verified that TPMT is responsible for the methylation of tellurite and methanetellurol to form methanetellurol and dimethyltelluride, respectively, and INMT is responsible for the methylation of methanetellurol and dimethyltelluride to form dimethyltelluride and trimethyltelluronium ion, respectively. These methylated compounds are typically detected in the urine and blood of animals that have ingested tellurite (Ogra et al. 2007, 2008). Furthermore, TMPT was identified as a tellurite-resistant gene in bacterial species (Cournoyer et al. 1998). We also demonstrated that TPMT gene knockdown increases the sensitivity to tellurite in human hepatoma HepG2 cells (Fukumoto et al. 2020). Therefore, trimethyltelluronium ion could be less toxic than tellurite, and both TPMT and INMT would contribute to reducing the toxicity of tellurite.

## Conclusions

We investigated the involvement of methyltransferases TPMT and INMT in the methylation of Te compounds using LC-ICP-MS and GC-MS and found that TPMT is involved in the methylation of tellurite and methanetellurol to form methanetellurol and dimethyltelluride, respectively. In addition, INMT catalyzes the methylation of methanetellurol and dimethyltelluride to produce dimethyltelluride and trimethyltelluronium ions, respectively. We identified the mechanism by which these two enzymes cooperatively generate the excreted form of Te observed in the urine of mammals (i.e., trimethyltelluronium ion). The fact that a similar reaction occurs using selenite suggests that the detoxification pathways of Te and Se oxyanions can antagonize each other.

## Supplementary Information

Below is the link to the electronic supplementary material.

Supplementary file1 (DOCX 605 KB)

## Data Availability

Data will be made available on request.
