# Supplementary material for: Thiopurine S‑methyltransferase- and indolethylamine N‑methyltransferase-mediated formation of methylated tellurium compounds from tellurite
Source: Arch Toxicol. 2024 Oct 17;99(1):237–44. doi: 10.1007/s00204-024-03890-4 (PMC11742336; doi:10.1007/s00204-024-03890-4)
Supplement: Supplementary file 1 — Supplementary file1 (DOCX 605 KB) [file 204_2024_3890_MOESM1_ESM.docx]

**Supplemental Information for (ATOX-D-24-00931)**

**Thiopurine *S*‑methyltransferase and indolethylamine *N*‑methyltransferase mediated formation of methylated tellurium compounds from tellurite**

Yu-ki Tanaka^a^, Ayuka Takata^a^, Karin Takahashi^a^, Yoshikazu Yamagishi^b^, Yasunori Fukumoto^a^, Noriyuki Suzuki^a†^, and Yasumitsu Ogra^a,*^

^a^ Graduate School of Pharmaceutical Sciences, Chiba University, 1-8-1 Inohana, Chuo, Chiba 260-8675, Japan

^b^ Graduate School of Medicine, Chiba University, 1-8-1 Inohana, Chuo, Chiba 260-8675, Japan

†Present affiliation and address: Faculty of Pharmaceutical Sciences, Toho University, 2-2-1 Miyama, Funabashi, Chiba 274-8510, Japan

**Corresponding author**

Yasumitsu Ogra

^*^ Tel/Fax: +81 43 226 2944 / E-mail: ogra@chiba-u.jp

**Table of Contents**

p. S2…Fig. S1. Elution profiles of tellurite and tellurate

p. S3…Fig. S2. Peak shapes of methanetelluronic acid (M_1_) and dimethyl telluroxide (M_2_) detected around the retention time of 1.8 min

p. S4…Fig. S3. Michaelis-Menten plots of methylation reaction catalyzed by TPMT using (a) potassium tellurite and (b) dimethyl ditelluride as substrate

p. S5…Fig. S4. Elution profiles of the reaction mixture of tellurite and TPMT with/without glutathione (GSH)

**Fig. S1.** Elution profiles of tellurite and tellurate

“Tellurite+H_2_O_2_” sample was reacted with an enzyme-free reaction buffer consisting of 1.0 μM SAM, 10 mM reduced glutathione, and 20 mM sodium phosphate buffer for 24 hours. Afterward, the solution was treated with hydrogen peroxide and catalase. The elution profile of the “Tellurite+H_2_O_2_” sample was markedly different from that of the original tellurite solution in which almost no peak was detected. On the other hand, the elution profile of the "Tellurite+H_2_O_2_" sample resembled that of the intact tellurate solution. Furthermore, when "Tellurite+H_2_O_2_" solution was spiked with tellurate, the resulting peak coincided with that of tellurate.

**Fig. S2.** Peak shapes of methanetelluronic acid (M1) and dimethyl telluroxide (M2) detected around the retention time of 1.8 min

Signal intensity is normalized to the maximum intensity between 1.5 and 2.5 min. After the enzymatic reaction of tellurite with TPMT, the peak became sharper and slightly shifted to an earlier retention time relative to that of methanetelluronic acid.

**Fig. S3.** Michaelis-Menten plots of methylation reaction catalyzed by TPMT using (a) potassium tellurite and (b) dimethyl ditelluride as substrate

**Fig. S4.** Elution profiles of the reaction mixture of tellurite and TPMT with/without glutathione (GSH)
